# Early-Age Evolution of Strength, Stiffness, and Non-Aging Creep of Concretes: Experimental Characterization and Correlation Analysis

**DOI:** 10.3390/ma12020207

**Published:** 2019-01-09

**Authors:** Mario Ausweger, Eva Binder, Olaf Lahayne, Roland Reihsner, Gerald Maier, Martin Peyerl, Bernhard Pichler

**Affiliations:** 1 Institute for Mechanics of Materials and Structures, TU Wien—Vienna University of Technology, Karlsplatz 13/202, 1040 Vienna, Austria; mario.ausweger@gmail.com (M.A.); eva.binder@tuwien.ac.at (E.B.); olaf.lahayne@tuwien.ac.at (O.L.); roland.reihsner@tuwien.ac.at (R.R.); 2 College of Civil Engineering, Tongji University, No. 1239, Si Ping Rd., Shanghai 200092, China; 3 Smart Minerals GmbH, Franz-Grill-Straße 9, 1030 Vienna, Austria; maier@smartminerals.at (G.M.); peyerl@smartminerals.at (M.P.)

**Keywords:** *fib* Model Code 2010, hardening of concrete, strength, stiffness, creep modulus

## Abstract

Six different concretes are characterized during material ages between 1 and 28 days. Standard tests regarding strength and stiffness are performed 1, 3, 7, 14, and 28 days after production. Innovative three-minute-long creep tests are repeated hourly during material ages between one and seven days. The results from the standard tests are used to assess and to improve formulas of the *fib* Model Code 2010: the correlation formula between the 28-day values of the strength and the stiffness, and the evolution formulas describing the early-age evolution of the strength and the stiffness during the first four weeks after production. The results from the innovative tests are used to develop a correlation formula between the 28-day values of Young’s modulus and the creep modulus, and an evolution formula describing the early-age evolution of the creep modulus during the first four weeks after production. Particularly, the analyzed CEM I concretes develop stiffness and strength significantly faster than described by the formulas of the Model Code. The creep modulus of the investigated concretes evolves significantly slower than their strength and stiffness. Thus, concrete loaded at early ages is surprisingly creep active, even if the material appears to be quite mature in terms of its strength and stiffness.

## 1. Introduction

Prestressed concrete construction and balanced cantilever construction are two examples of an entire class of construction methods, in which reinforced concrete structures are loaded already well *before* they reach an age of 28 days. Related design calculations require quantitative knowledge of mechanical properties of concrete at early ages. The *fib* Model Code 2010 [1] provides formulas for early-age strength and stiffness values of concrete, as a function of the uniaxial compressive strength reached 28 days after production, $f_{c,28d}$. The evolution formula describing the early-age evolution of the uniaxial compressive strength during the first four weeks after production reads as [1]

(1) $$f_{c}\left(t\right)=f_{c,28d}\;\exp\left[s\left(1-\sqrt{\frac{28\;\mathrm{days}}{t}}\right)\right]\;.$$

In Equation (1), the dimensionless parameter *s* is related to the speed with which the 28-day strength is approached: the smaller *s*, the faster is the early-age strength evolution, and vice versa. Notably, *s* depends on $f_{c,28d}$ and the type of cement used to produce the concrete of interest (see Table 1).

The correlation formula that allows for quantifying the 28-day value of Young’s modulus of concrete, $E_{u,28d}$, based on knowledge regarding $f_{c,28d}$ reads as [1]

(2) $$E_{u,28d}=21.5\;\mathrm{GPa}\cdot \alpha {\left(\frac{f_{c,28d}}{10\;\mathrm{MPa}}\right)}^{0.\dot{3}}\;.$$

In Equation (2), the dimensionless parameter α accounts for the stiffness of the aggregates used to produce the concrete of interest (see Table 1). The evolution formula describing the early-age evolution of Young’s modulus during the first four weeks after production, reads as [1]

(3) $$E_{u}\left(t\right)=E_{u,28d}{\left\{\exp\left[s\left(1-\sqrt{\frac{28\;\mathrm{days}}{t}}\right)\right]\right\}}^{0.5}\;.$$

Equations (1)–(3) refer to concrete curing at 20 ${}^{\circ }$C.

In the present paper, standard and innovative test methods are combined in order to characterize the evolution of the strength, the stiffness, and the creep activity of a set of contemporary concretes, with two aims: (i) to assess and to improve the reliability of the Equations (1)–(3), and (ii) to develop similar formulas for quantification of the early-age evolution of the creep activity of concrete. Thereby, the following two aspects provide the underlying motivation:

1. The cement and concrete industry makes efforts to reduce the emission of CO${}_{2}$ associated with the production of their binders [2]. Thus, commercially marketed cements and the corresponding mix designs of concrete experienced a considerable development [3]. This raises the question concerning the reliability of Equations (1)–(3) for contemporary concretes.
2. The serviceability of concrete structures which were built decades ago and loaded, at that time, *at early ages*, is frequently challenged by unexpectedly large creep deformation [4]. This raises the question concerning the evolution of the creep activity of contemporary concretes at early ages.

As for the experimental determination of the compressive strength of concrete, standards such as the Eurocode [5] suggest tests on cubes because they are simple to perform. The obtained strength values are by some 20% larger than the strength values obtained with cylinders (see e.g., refs. [6,7,8]). This is because shear stresses are activated by friction in the interfaces between the specimens and the load platens. The self-equilibrated shear stresses affect the entire tested specimen [9]. They result in a confinement of the material, which increases the strength of the tested cube. The uniaxial compressive strength of concrete is, at least in good approximation, accessible based on tests on specimens with a height-to-width ratio of 2. This is to be understood based on the principle of Saint Venant [10] (see also [11,12,13]). It implies that the self-equilibrated shear stresses decrease with increasing distance from the interfaces between the specimen and the load platens, such that they reach insignificant magnitudes in a distance amounting to roughly one times the characteristic in-plane dimension of the interfaces between the specimen and the load platens (see [9] for validation of this statement by means of Finite Element simulations). Thus, the central part of a cylinder with a width of 150 mm and a height of 300 mm may be considered to be virtually free of undesired shear stresses.

As for the experimental determination of the stiffness of concrete, several testing strategies are commonly used. Standards typically recommend to quantify the unloading modulus in uniaxial compression tests with the maximum load amounting to one-third of the expected compressive strength (see, e.g., the Austrian standard [14]). As for characterization of the early-age stiffening of concrete, testing has to be repeated frequently (see, e.g., refs. [15,16,17,18,19,20] for comprehensive early-age testing campaigns). As for early-age characterization since extremely early ages, starting at setting of the material, it is popular to determine the so-called dynamic stiffness of concrete based on ultrasonic tests (see, e.g., refs. [21,22,23,24,25]). An elegant resonance frequency method based on very small vibrations of beam-like concrete specimens hardening inside a flexible mold was developed by Azenha et al. [26,27,28]. A test method that allows for carrying out compression test inside a temperature-controlled mold was developed by Boulay et al. [29,30]. The described early-age testing campaigns have stimulated pan-European modeling activities (see, e.g., refs. [31,32]).

As for the experimental determination of the creep activity of concrete at early ages, mostly aging creep tests are performed. “Aging” implies that the microstructure of concrete evolves during the test because the chemical reaction between the binder and the water continuously consumes these two constituents and results in the progressive formation of hydration products. Aging creep tests pose great challenges for multiscale modeling (see, e.g., refs. [33,34]). This was the motivation for the development of *nonaging* creep testing protocols. They consist of ultra-short creep tests with a duration of a few minutes only [17,25]. Although the hydration reaction is ongoing, it does not change the microstructure significantly during a few minutes, hence the terminology “nonaging” creep testing. This has allowed for developing predictive multiscale models, based on creep constants of microscopic hydrate-gel needles [17,35,36]. Rather recently, creep tests on so-called equivalent materials were carried out [37]. In these materials, part of the cement is replaced with a finely ground filler [38]. After complete hydration, this approach leads to non-aging specimens which are equivalent to specific early-age microstructures of real hydrating systems.

The present paper is structured as follows: Section 2 presents the results obtained from early-age testing campaigns on six different concretes. The evolution of their strength and stiffness is characterized following the Austrian standard [14]. The evolution of their viscoelastic properties is characterized by means of hourly-repeated three-minutes-long creep tests, carried out from one day to seven days after production, following the test protocol of Irfan-ul-Hassan et al. [17]. In Section 3, Equations (1)–(3) are both assessed and improved, and similar formulas for quantification of the early-age evolution of the creep activity of concrete are developed. The results are discussed in Section 4. Conclusions are drawn in Section 5.

## 2. Early-Age Characterization of Strength, Stiffness, and Creep Properties

### 2.1. Materials

The six tested concretes refer to three different strength classes (see Table 2). They are made of three different types of cement and three different initial water-to-cement mass ratios, w/c, as well as two types of rounded aggregates: quartz, representative for western Austria, and limestone, representative for eastern Austria. The mass densities of these aggregates amount to 2.65 g/cm${}^{3}$ and to 2.72 g/cm${}^{3}$, respectively. The concretes are designed to be frost-thaw resistant, in an environment with moderate water saturation and exposed to de-icing agents. Because of these additional requirements, it was decided to produce the CEM I-based C40/50 with a CEM I 52.5. An air-entraining agent is added in order to obtain air contents in the range from range 2% to 6%. The mix proportions are listed in Table 2.

### 2.2. Standard Testing of Strength and Stiffness Following the Austrian Standard

The early-age evolution of the compressive strength and the unloading modulus was characterized by means of standard tests in the laboratory of the Smart Minerals GmbH. The experiments were carried out at material ages amounting to 1, 3, 7, 14, and 28 days after production following the Austrian standard [14].

Following a standard protocol used in the engineering practice, the compressive strength was determined based on destructive compression tests on concrete *cubes* with side-lengths amounting to 15 cm. The formulas of the *fib* Model Code 2010 [1], however, refer to the uniaxial compressive strength obtained on cylinders (see, e.g., Equation (1)). Thus, experimentally determined values of the cube compressive strength must be translated into equivalent values of the cylinder compressive strength. This is done in agreement with the recommendations of the *fib* Model Code 2010 [1], i.e., the cylinder compressive strength values were estimated by dividing the cube compressive strength values by 1.2. For each of the six concretes, for each of the two investigated air contents, and for each of the five material ages of interest, two specimens were crushed. Thus, in total, 6×2×5×2=120 strength tests were carried out.

The unloading modulus was determined based on non-destructive loading-unloading cycles on concrete prisms with dimensions of 10 cm × 10 cm × 36 cm. The unloading modulus was quantified as the quotient of the stress and strain differences between load levels amounting to 1/3 and 1/30 of the uniaxial compressive strength. For each of the 6 concretes and for each of the 2 investigated air contents, 1 specimen was produced. Each of the resulting 6×2×1=12 specimens were subjected, at 5 material ages of interest, to nondestructive tests. Thus, in total 12×5=60 individual stiffness test were carried out.

Storage and testing were carried out at quasi-isothermal conditions of $20\pm 5{\;}^{\circ }$C. The specimens remained in their molds for 24±2 h. Afterwards, the concrete cubes were covered by several layers of food preservation foil in order to avoid drying. The concrete prisms, in turn, were stored after demolding under water until testing.

**Figure 1:**
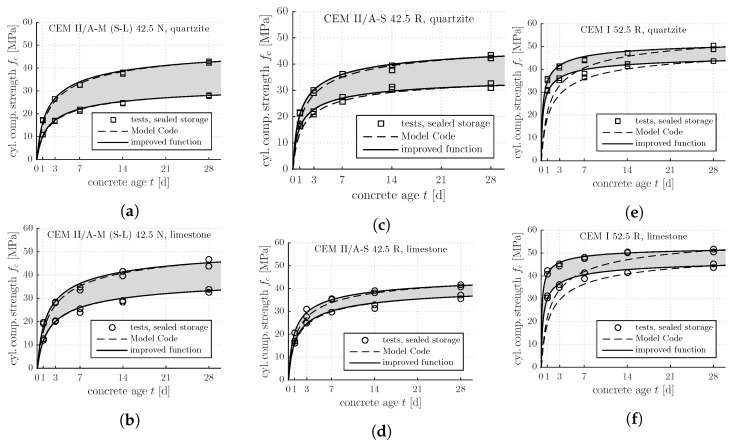
Results from standard testing following the Austrian standard [14]: early-age evolution of the cylinder compressive strength of the concretes listed in Table 2; the upper and the lower boundaries of the gray-shaded areas refer to air contents amounting to 2% and 6%, respectively: (**a**) CEM II/A-M (S-L) 42.5 N, quartzite; (**b**) CEM II/A-M (S-L) 42.5 N, limestone; (**c**) CEM II/A-S 42.5 R, quartzite; (**d**) CEM II/A-S 42.5 R, limestone; (**e**) CEM I 52.5 R, quartzite; (**f**) CEM I 52.5 R, limestone.

**Figure 2:**
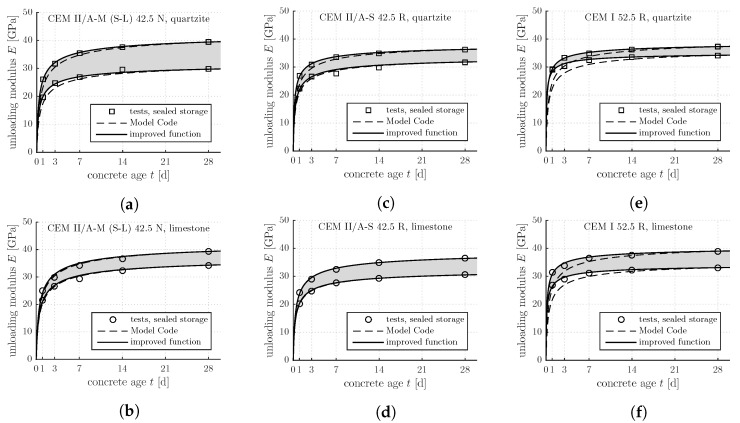
Results from standard testing following the Austrian standard [14]: early-age evolution of the unloading modulus of the concretes listed in Table 2; the upper and the lower boundaries of the gray-shaded areas refer to air contents amounting to 2% and 6%, respectively: (**a**) CEM II/A-M (S-L) 42.5 N, quartzite; (**b**) CEM II/A-M (S-L) 42.5 N, limestone; (**c**) CEM II/A-S 42.5 R, quartzite; (**d**) CEM II/A-S 42.5 R, limestone; (**e**) CEM I 52.5 R, quartzite; (**f**) CEM I 52.5 R, limestone.

The obtained experimental results are shown in Figure 1 and Figure 2. Squares refer to quartz aggregates, circles to limestone. The data points in Figure 2 represent average values resulting from three loading-unloading cycles which were carried out immediately one after the other.

### 2.3. Innovative Testing of Stiffness and Creep Properties According to the Protocol Developed

**Figure 3:**
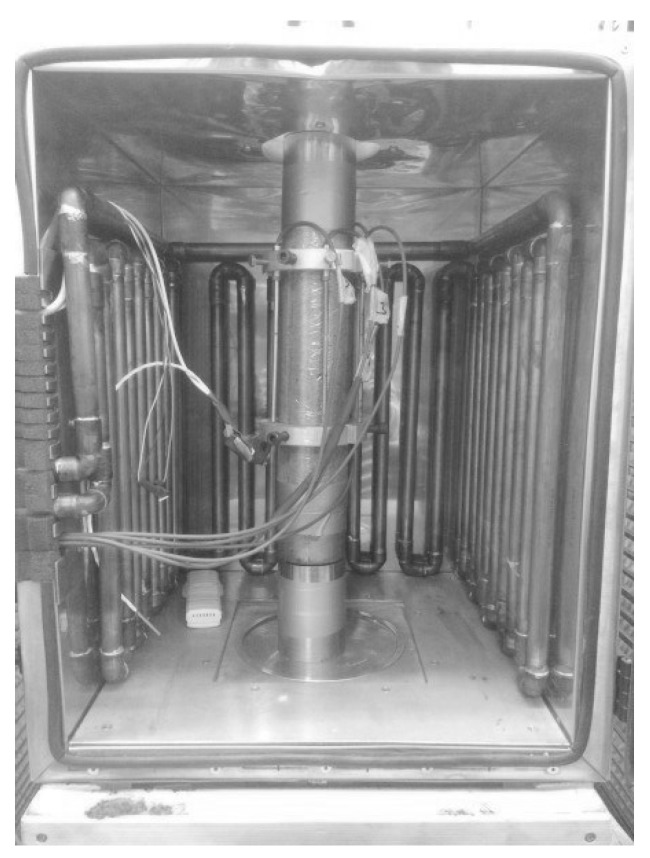
Test setup inside a temperature chamber containing two temperature sensors and copper pipes filled with conditioning fluid: two aluminum rings are attached to the concrete specimen, holding five displacement sensors; after [17].

The characterization of early-age stiffness and creep properties follows the protocol of Irfan-ul-Hassan et al. [17]. This includes hourly-repeated three-minutes-long creep tests under uniaxial compression. The tests are carried out on concrete cylinders with a diameter of 7 cm and an axial length of 30 cm (see Figure 3).

Specimens are demolded 23 h after production. They are covered by several layers of food preservation foil, in order to protect them from drying. The first test is carried out 24 h after production. Hourly testing is continued up to material ages of seven days to eight days, depending on the availability of the testing machine. Thus, every specimen undergoes a series of 144 to 168 three-minutes-long creep tests. In order to ensure non-destructive testing, the maximum load is restricted to 20% of the strength reached at the time of testing (see Figure 1). The tests are carried out under force control. At first, the desired load level is approached with a loading speed of 7.697kN/s, equivalent to a stress rate of 2MPa/s, following Irfan-ul-Hassan et al. [17]. This speed of loading is (i) small enough as to ensure that a quasi-static test is carried out (see [39]), and (ii) fast enough as to ensure that the duration of the loading phase is by two orders of magnitude smaller compared to the following creep test, during which the desired load level is kept constant for 180 seconds. Finally, unloading is carried out with a speed of 3.849kN/s, equivalent to a stress rate of 1MPa/s. For each of the six concretes, four specimens were produced. Each of the resulting 6×4=24 specimens were subjected, at 144 to 168 material ages of interest, to three-minutes-long tests. Thus, in total, some 4000 individual creep tests were carried out.

The applied forces and the changes of length of the specimens are measured as follows. The forces are measured by the load cell integrated in the used testing machine, which is of type Walter and Bai LFM 150, operated under the control of the software "test Xpert" of Zwick/Roell. The changes of length are measured directly at the specimens, using five inductive displacement sensors of Hottinger Baldwin type. The measurement signals are recorded with a frequency of 100 Hz. The stresses σ(t) are obtained by dividing the measured force values, F(t), by the nominal cross-sectional area $A=3849\;{\mathrm{mm}}^{2}$:

(4) $$\sigma \left(t\right)=\frac{F(t)}{A}\;.$$

The strains $\varepsilon_{exp}\left(t\right)$ are obtained by dividing the averaged readings of the five displacement sensors by the measurement length $\ell_{0}=164\;\mathrm{mm}$:

(5) $$\varepsilon_{exp}\left(t\right)=\frac{1}{5}\sum_{i=1}^{5}\frac{\Delta \ell_{i}\left(t\right)}{\ell_{0}}\;\;,$$

where $\Delta \ell_{i}\left(t\right)$ denotes the change of length measured at time instant *t* by the $i^{\mathrm{th}}$ displacement sensor.

From the measurement data of each three-minute-long creep test, the elastic Young’s modulus *E* and the creep modulus $E_{c}$ are identified by minimizing the difference between the measured strain evolution, $\varepsilon_{exp}\left(t\right)$, and the modeled strain evolution, $\varepsilon_{mod}\left(t\right)$:

(6) $$E=\sqrt{\frac{1}{N}\sum_{i=1}^{N}{\left[\varepsilon_{exp}\left(t_{i}\right)-\varepsilon_{mod}\left(t_{i}\right)\right]}^{2}}\to \min\;,$$

where *N* stands for the total number of experimental readings considered for test evaluation, recorded during the phase of application of the load and the subsequent phase of constant loading, typically amounting to *N* = 18,000.

Modeling is based on the theory of viscoelasticity, considering that the deformation during the short loading phase contains both elastic *and* time-dependent contributions [17]. In more detail, the modeled strain evolution is computed based on Boltzmann’s superposition principle, formulated in terms of the following convolution integral [40]

(7) $$\varepsilon_{mod}\left(t\right)=\int_{0}^{t}J\left(t-\tau \right)\;\frac{d\sigma }{d\tau }\;d\tau \;.$$

In Equation (7), J(t−τ) denotes the uniaxial creep function, and dσ/dτ stands for the time-derivative of the stress history. As for the creep function, the following power law is used by analogy to [17,35,41]:

(8) $$J\left(t-\tau \right)=\frac{1}{E}+\frac{1}{E_{c}}{\left(\frac{t-\tau }{t_{ref}}\right)}^{\beta }\;,$$

where $t_{ref}$ = 86,400 s and β=0.25 stand for a reference time and the creep exponent, respectively. The stress increases linearly during the application of the loading, in the time interval $0\leq t<t_{*}$. Thus, the stress rate is constant. It is denoted as $\dot{\sigma }$. During the subsequent 180 s of constant loading, in the time interval $t_{*}<t\leq \left(t_{*}+180\;s\right)$, the stress rate vanishes:

(9) $$\frac{d\sigma }{d\tau }=\left\{\begin{array}{c} \dot{\sigma }=\mathrm{const}.\cdots \;0\leq t<t_{*},\\ \dot{\sigma }=0\cdots \;t_{*}<t\leq \left(t_{*}+180\;s\right). \end{array}\right.$$

The convolution integral in Equation (7) can be solved in a piecewise analytical fashion. As for the loading phase, Equation (7) is specialized for $0<t\leq t_{*}$. Inserting Equations (8) and (9) into the resulting expression yields

(10) $$\varepsilon_{mod}\left(t\right)=\frac{\sigma (t)}{E}+\frac{\dot{\sigma }\;t_{ref}}{E_{c}\;\left(\beta +1\right)}{\left(\frac{t}{t_{ref}}\right)}^{\beta +1}\;0\leq t\leq t_{*}\;.$$

As for the load plateau, Equation (7) is specialized for $t_{*}<t\leq \left(t_{*}+180\;s\right)$. Inserting Equations (8) and (9) into the resulting expression yields

(11) $$\varepsilon_{mod}\left(t\right)=\frac{\Delta \sigma }{E}+\frac{\dot{\sigma }\;t_{ref}}{E_{c}\;\left(\beta +1\right)}\left[{\left(\frac{t}{t_{ref}}\right)}^{\beta +1}-{\left(\frac{t-t_{*}}{t_{ref}}\right)}^{\beta +1}\right]\;t_{*}\leq t\leq \left(t_{*}+180\;s\right)\;\;,$$

where Δσ denotes the total stress increment imposed during loading.

**Figure 4:**
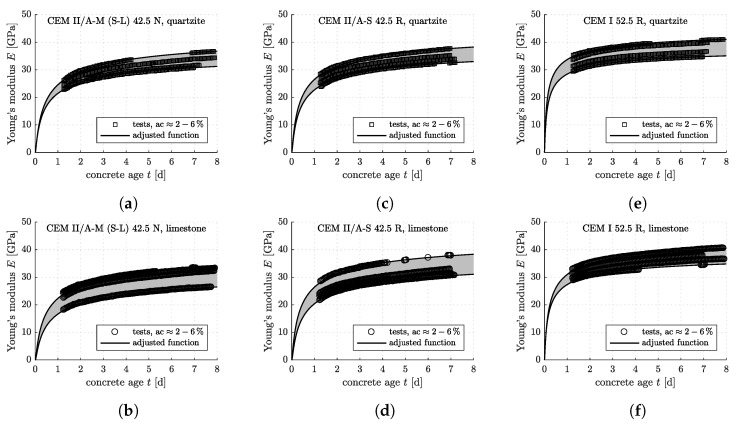
Results from innovative testing according to the protocol developed in [17]: early-age evolution of the elastic Young’s modulus of the concretes listed in Table 2, with nominal air contents from 2% to 6%: (**a**) CEM II/A-M (S-L) 42.5 N, quartzite; (**b**) CEM II/A-M (S-L) 42.5 N, limestone; (**c**) CEM II/A-S 42.5 R, quartzite; (**d**) CEM II/A-S 42.5 R, limestone; (**e**) CEM I 52.5 R, quartzite; (**f**) CEM I 52.5 R, limestone.

**Figure 5:**
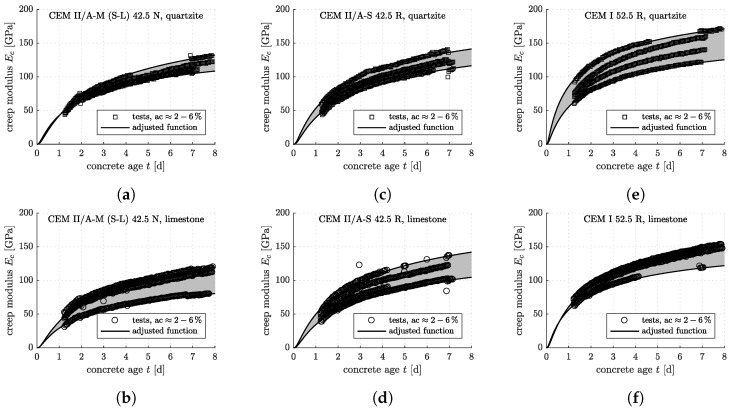
Results from innovative testing according to the protocol developed in [17]: early-age evolution of the creep modulus of the concretes listed in Table 2, with nominal air contents from 2% to 6%: (**a**) CEM II/A-M (S-L) 42.5 N, quartzite; (**b**) CEM II/A-M (S-L) 42.5 N, limestone; (**c**) CEM II/A-S 42.5 R, quartzite; (**d**) CEM II/A-S 42.5 R, limestone; (**e**) CEM I 52.5 R, quartzite; (**f**) CEM I 52.5 R, limestone.

The optimization problem defined in Equation (6) is solved iteratively, with progressively refined search grids, until the global minimum of the objective function is found (see [17] for details). The described optimization procedure is applied to each individual three-minute creep test, resulting in some 4000 sets of *E* and $E_{c}$ (see Figure 4 and Figure 5 for the results).

## 3. Results, Interpretation, and Correlation

### 3.1. Assessment and Improvement of Formulas of the fib Model Code 2010

Equations (1) and (3), describing the evolution of the strength and the stiffness of concrete during the first four weeks after production, are assessed based on the experimental data shown in Figure 1 and Figure 2. Thereby, $f_{c,28d}$ and $E_{u,28d}$ are set equal to the corresponding test results. The dimensionless parameters *s* and α are taken from Table 1. As for concretes produced with CEM II cements, the evolution Formulas (1) and (3) are quite reliable, compare the punctiform symbols with the dashed curves in Figure 1a–d and Figure 2a–d. As for concretes produced with the CEM I cement, the evolution Formulas (1) and (3) significantly underestimate the early-age evolution of the strength and the stiffness, compare the punctiform symbols with the dashed curves in Figure 1e–f and Figure 2e–f.

The reliability of Equations (1) and (3) can be increased by adjusting the values of the dimensionless *s*-parameter. To this end, *s* is replaced by $s_{f_{c}}$ in Equation (1) and by $s_{E}$ in Equation (3). Both $s_{f_{c}}$ and $s_{E}$ are optimized such that the squared differences between measurement strength or stiffness values $y(t_{i})$ and corresponding calculated values $f(t_{i},s)$ attain a minimum:

(12) $$\sum_{i=1}^{n}{\left[f\left(t_{i},s\right)-y\left(t_{i}\right)\right]}^{2}\;\to \;\min\;.$$

For each of the three analyzed types of concrete, an average value $\bar{s}$ is computed based on four optimal $s_{f_{c}}$ values and four optimal $s_{E}$ values (see the last column of Table 3). As for the CEM II concretes, optimal $\bar{s}$ values are by some 10% smaller than the values recommended by the *fib* Model Code 2010, compare Table 1 and Table 3 (see also the solid lines in Figure 1a–d and Figure 2a–d). As for the CEM I concretes, in turn, optimal $\bar{s}$ values are by more than a factor of 2 smaller than the values recommended by the *fib* Model Code 2010, compare Table 1 and Table 3 (see also the solid lines in Figure 1e–f and Figure 2e–f).

**Figure 6:**
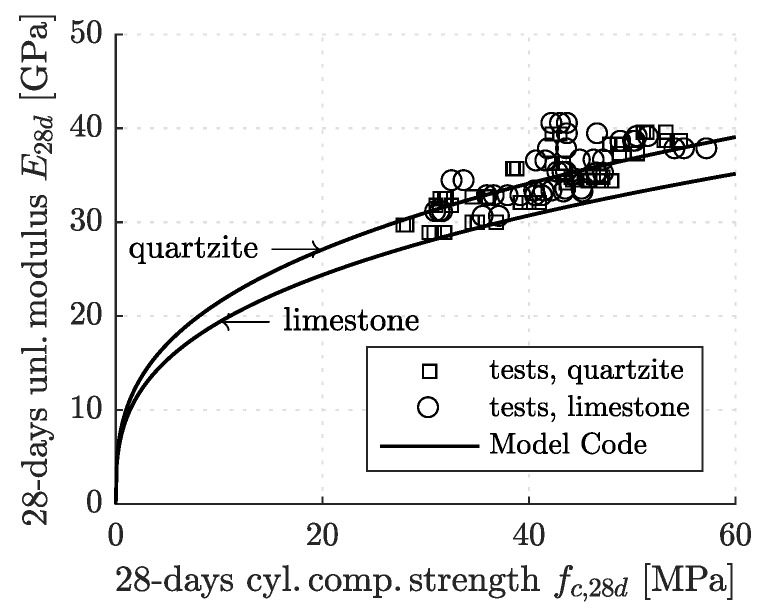
Correlation between 28-day values of the cylinder compressive strength and the unloading modulus: the points refer to experimental data from standard testing of strength and stiffness following the Austrian standard [14]; the graphs refer to Equation (3).

Equation (2), describing the correlation between $f_{c,28d}$ and $E_{u,28d}$ is assessed based on measurements obtained 28 days after production. The test data form a quite dense cloud of points, regardless of the air content and the types of cement and aggregates (see the punctiform symbols in Figure 6). This is interesting because the *fib* Model Code 2010 expects that concretes made with limestone aggregates have smaller Young’s moduli than equally strong concretes made with quartz aggregates (see the two graphs in Figure 6).

The reliability of Equation (2) can be increased by adjusting the values of the dimensionless α-parameter. The optimal value regarding quartz-based concretes is equal to the value recommended by the *fib* Model Code 2010: $\alpha_{opt}=\alpha =1.0$. Independent from that, the optimal value regarding limestone-based concretes is obtained as $\alpha_{opt}=1.0$. This suggests that no distinction between quartz and limestone aggregates is necessary when it comes to the correlation between $f_{c,28d}$ and $E_{u,28d}$.

### 3.2. Development of fib Model Code 2010-Inspired Formulas for the Creep Modulus

The following developments aim at deriving a correlation formula between the 28-day values of the elastic Young’s modulus and the creep modulus, and an evolution formula that describes the development of the creep modulus during the first four weeks after production, such that the 28-day value is reached in the end.

Because of time constraints, hourly testing had to be stopped seven to eight days after production of the specimens. In order to estimate 28-day values of *E* and $E_{c}$, the test results are extrapolated to material ages of 28 days. To this end, the test results are fitted based on the following formulas:

(13) $$E\left(t\right)=E_{8d}\cdot {\left\{\exp\left[s_{E,8d}\cdot \left(1-\sqrt{\frac{8\;\mathrm{days}}{t}}\right)\right]\right\}}^{0.5},$$

(14) $$E_{c}\left(t\right)=E_{c,8d}\cdot {\left\{\exp\left[s_{E_{c},8d}\cdot \left(1-\sqrt{\frac{8\;\mathrm{days}}{t}}\right)\right]\right\}}^{0.5},$$

where $E_{8d}$ and $E_{c,8d}$ denote values of Young’s modulus and creep modulus referring to material ages amounting to eight days. Together with the dimensionless parameters $s_{E,8d}$ and $s_{E_{c},8d}$, they are determined such that test results are reproduced in the best possible fashion (see Table 4 and Table 5).

Estimated 28-day values of $E_{28d}$ and $E_{c,28d}$ are obtained by evaluating the optimized functions for t=28days (see Table 6).

**Figure 7:**
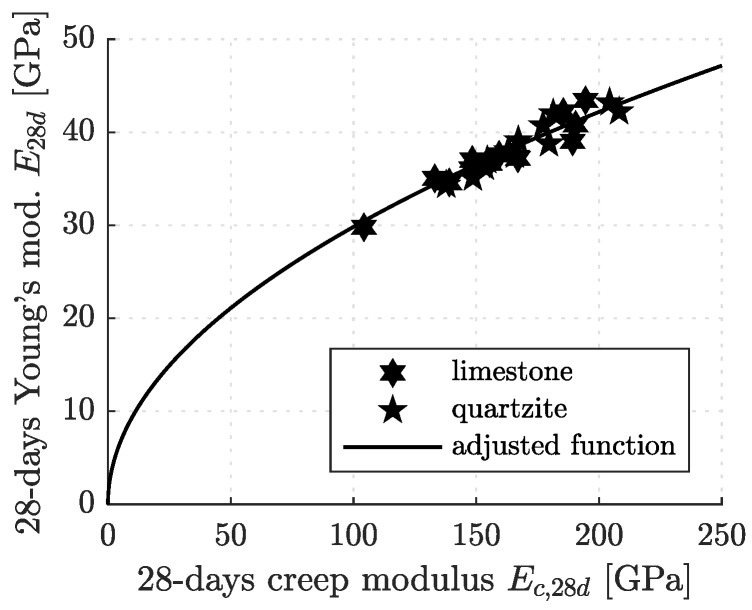
Correlation between 28-day values of the elastic Young’s modulus and the creep modulus: the points refer to extrapolated values from innovative testing of stiffness and creep according to the protocol developed in [17] (see Table 5 and Table 6); the graph refers to Equation (15).

Marking these values in a diagram showing $E_{28d}$ as a function of $E_{c,28d}$ (see Figure 7), a dense data cloud is obtained, irrespective of the air content and the types of cement and aggregates used.

The following correlation function approximates this data cloud reliably:

(15) $$E_{c,28d}=51.9\;\mathrm{GPa}\cdot {\left(\frac{E_{28d}}{21.5\;\mathrm{GPa}}\right)}^{2}.$$

See the solid line in Figure 7. In order to establish a correlation formula between $E_{c,28d}$ and $f_{c,28d}$, Equation (2) is inserted into Equation (15). This yields

(16) $$E_{c,28d}=51.9\;\mathrm{GPa}\cdot {\left(\alpha \cdot \frac{f_{c,28d}}{10\;\mathrm{MPa}}\right)}^{2/3}.$$

Equation (16) allows for quantifying the 28-day value of the creep modulus based on the 28-day value of the uniaxial compressive strength.

Finally, an evolution formula is developed, which describes the early-age evolution of the creep modulus during the first four weeks after production, such that the 28-day value is reached in the end:

(17) $$E_{c}\left(t\right)=E_{c,28d}\cdot {\left\{\exp\left[s_{E_{c}}\cdot \left(1-\sqrt{\frac{28\;\mathrm{days}}{t}}\right)\right]\right\}}^{0.5}.$$

The dimensionless parameter $s_{E_{c}}$ is optimized, based on the values of the creep modulus, experimentally determined during the first week after concrete production (see Table 7). The corresponding mean value ${\bar{s}}_{E_{c}}$ is computed for each of the three classes of concrete (see the last column of Table 7).

## 4. Discussion

**Figure 8:**
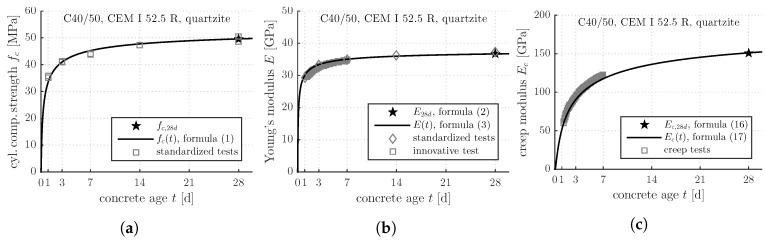
Exemplary application of the evolution and correlation Formulas (1)–(3), (16) and (17) and comparison with test data: early-age evolutions of (**a**) the cylinder compressive strength; (**b**) Young’s modulus; and (**c**) the creep modulus.

The improved and the newly developed formulas allow for quantifying the early-age evolutions of the strength, the stiffness, and the creep activity of concretes, based on knowledge regarding the 28-day values of the uniaxial compressive strength, $f_{c,28d}$:

- Inserting $f_{c,28d}$ together with the improved value of the *s*-parameter (see Table 3), into the evolution Formula (1), allows for quantifying the early-age evolution of the uniaxial compressive strength (see the solid line in Figure 8a).
- Inserting $f_{c,28d}$ together with the improved value of the α-parameter ($\alpha_{opt}=1.0$ both for quartz and limestone) into the correlation Formula (2) yields $E_{28d}$, the 28-day value of Young’s modulus (see the star symbol in Figure 8b).
- Inserting $E_{28d}$ together with the improved value of the *s*-parameter (see Table 3), into the evolution Formula (3), allows for quantifying the early-age evolution of Young’s modulus (see the solid line in Figure 8b).
- Inserting $f_{c,28d}$ together with the improved value of the α-parameter ($\alpha_{opt}=1.0$ both for quartz and limestone) into the newly developed correlation Formula (16) yields $E_{c,28d}$, the 28-day value of the creep modulus (see the star symbol in Figure 8c).
- Inserting $E_{c,28d}$ together with the newly introduced $s_{E_{c}}$-parameter (see Table 7), into the evolution Formula (17), allows for quantifying the early-age evolution of the creep modulus (see the solid line in Figure 8c).

The presented study underlines that correlations between the 28-day values of the compressive strength and Young’s modulus (see Figure 6) and between Young’s modulus and the creep modulus (see Figure 7), are *universal* for all tested concretes, irrespective of their composition, defined in terms of type of binder, type of aggregates, and air content. As for the early-age evolutions of the strength, the stiffness, and the creep properties, it is of prime importance (i) to know the 28-day value of the mechanical property of interest, and (ii) to determine the dimensionless $\bar{s}$-parameter (see Table 3) and ${\bar{s}}_{E_{c}}$-parameter (Table 7), respectively, for the type of binder used to produce concrete. Detailed knowledge regarding the type of aggregates and the air content turned out to be of minor importance, at least for the concretes analyzed in the presented study.

**Figure 9:**
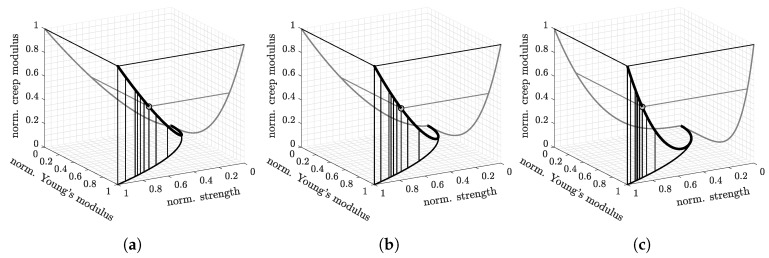
Correlation between normalized values of the cylinder compressive strength, Young’s modulus, and the creep modulus, quantified based on the evolution and correlation Formulas (1)–(3), (16) and (17): (**a**) CEM II/A-M (S-L) 42.5 N; (**b**) CEM II/A-S 42.5 R; (**c**) CEM I 52.5 R (see also Table 8).

The experimental results underline (i) that Young’s modulus increases faster than the strength, and (ii) that the strength increases significantly faster than the creep modulus (see Figure 9). The CEM I concretes, for instance, reached three days after production a Young’s modulus amounting to 91% of $E_{28d}$, a uniaxial compressive strength amounting to 83% of $f_{c,28d}$, and a creep modulus amounting to only 60% of $E_{c,28d}$ (see Table 8 for all studied concretes and other material ages).

In addition, stiffness and strength of CEM I concretes evolve faster compared to CEM II concretes, whereas the creep modulus of CEM I concretes increases slower compared to CEM II concretes (see also Figure 9).

## 5. Conclusions

From the presented study, the following conclusions are drawn:

- Both the strength and the stiffness of the tested concretes evolved faster than expected based on the formulas of the *fib* Model Code 2010 (see Equations (1)–(3)).
- These formulas are *qualitatively* satisfactory. *Quantitatively*, they can be customized for contemporary concretes, simply by optimizing the *s*- and α-parameters based on early-age test data.
- As for CEM I and CEM II concretes, respectively, the optimized *s*-values are more than 50% and some 10% smaller than the values recommended by the *fib* Model Code 2010.
- The optimized α-values amount to 1.0 both for the used quartz and limestone aggregates. Thus, limestone aggregates which are representative for eastern Austria are stiffer than expected by the *fib* Model Code 2010.
- The newly developed formulas for the creep modulus follow the philosophy of the *fib* Model Code 2010. The correlation Formula (16) allows for quantifying the 28-day value of the creep modulus, $E_{c,28d}$ based on knowledge of the 28-day value of the uniaxial compressive strength. The evolution Formula (17) allows for quantifying the early-age evolution of the creep modulus during the first four weeks after production.
- Relative to values reached 28 days after production, the Young’s modulus increases faster than the uniaxial compressive strength, and the strength increases significantly faster than the creep modulus. Thus, concrete loaded at early ages is surprisingly creep active, even if the material appears to be quite mature in terms of its strength and stiffness.

As for future follow-up studies, it will be interesting to characterize concretes with basaltic or sandstone aggregates because their nominal α-values amount to 1.2 and 0.7, respectively.

## Figures and Tables

**Table 1:** Dimensionless parameters *s* and α according to *fib* Model Code 2010 [1] (see Equations (1)–(3)).

$f_{c,28d}$	Cement Type	*s*	Aggregate Type	α
≤60 MPa	32.5 N	0.38	basalt, dense limestone	1.2
≤60 MPa	32.5 R, 42.5 N	0.25	quartz	1.0
≤60 MPa	42.5 R, 52.5 N, 52.5 R	0.20	limestone	0.9
>60 MPa	all classes	0.20	sandstone	0.7

**Table 2:** Mix proportions of the six characterized concretes: masses of water, aggregates (Agg.), cement (Cem.), supplementary cementitious material (SCM), air-entraining agent (AEA), and super plasticizer (SP) for one cubic meter of concrete

				Masses for One Cubic Meter of Concrete					
**Concrete Type**	**Cement Type**	**Aggregate Type**	**w/b (-)**	**Water (kg)**	**Agg. (kg)**	**Cem. (kg)**	**SCM (kg)**	**AEA (kg)**	**SP (kg)**
C30/37/W55	CEM II/	quartz	0.48	170	1832	320	40	0.13–6.05	3.78
3-10B5/GK22/F52	A-M(S-L) 42.5 N	limestone	0.48	170	1875	320	40	0.10–4.70	4.42
C35/45	CEM II/	quartz	0.45	185	1755	410	-	1.03–7.59	0.62
3-10 B5/GK22/F52	A-S 42.5 R	limestone	0.45	185	1796	410	-	0.21–5.08	0.16
C40/50	CEM I/	quartz	0.42	175	1773	420	-	0.13-3.53	1.13
3-10 B5/GK22/F52	52.5 R	limestone	0.42	175	1814	420	-	0.17–5.84	6.17

**Table 3:** Improvement of Equations (1) and (3) describing the early-age evolution of the compressive strength and Young’s modulus: optimal values of the dimensionless parameters $s_{f_{c}}$ and $s_{E}$, as well as their mean values $\bar{s}$, identified based on strength and stiffness tests carried out following the Austrian standard [14].

Cement Type	Aggregate	$s_{f_{c}}$	$s_{E}$	$\bar{s}$
CEM II/A-M (S-L) 42.5 N	quartz	0.23/0.24	0.20/0.19	0.22
	limestone	0.22/0.25	0.23/0.23	
CEM II/A-S 42.5 R	quartz	0.17/0.16	0.15/0.18	0.18
	limestone	0.16/0.19	0.20/0.19	
CEM I 52.5 R	quartz	0.08/0.09	0.11/0.09	0.09
	limestone	0.05/0.09	0.11/0.11	

**Table 4:** Results obtained from fitting of the experimentally determined early-age evolutions of Young’s modulus based on Equation (13): optimal values of $E_{8d}$ and $s_{E,8d}$.

Cement Type	Aggregate	$E_{8d}$ [GPa]	$s_{E,8d}$
CEM II/A-M (S-L) 42.5 N	quartz	34.31/36.68/31.57/30.76	0.44/0.45/0.43/0.44
	limestone	32.22/33.32/33.22/26.36	0.48/0.48/0.42/0.52
CEM II/A-S 42.5 R	quartz	33.86/35.25/32.60/37.70	0.42/0.42/0.43/0.43
	limestone	32.92/37.81/30.67/31.34	0.49/0.44/0.49/0.47
CEM I 52.5 R	quartz	39.80/41.03/36.63/34.76	0.24/0.22/0.23/0.25
	limestone	37.88/40.50/36.50/34.51	0.29/0.30/0.28/0.28

**Table 5:** Results obtained from fitting of the experimentally determined early-age evolutions of the creep modulus based on Equation (14): optimal values of $E_{c,8d}$ and $s_{E_{c},8d}$.

Cement Type	Aggregate	$E_{c,8d}$ [GPa]	$s_{E_{c},8d}$
CEM II/A-M (S-L) 42.5 N	quartz	122.41/131.76/110.86/104.70	1.22/1.26/1.19/1.10
	limestone	111.77/115.09/119.76/79.68	1.19/1.31/1.11/1.13
CEM II/A-S 42.5 R	quartz	122.08/123.60/112.77/136.10	1.24/1.20/1.25/1.14
	limestone	121.95/136.57/101.07/101.53	1.25/1.23/1.29/1.08
CEM I 52.5 R	quartz	160.57/170.77/140.71/121.45	1.05/0.76/0.99/0.96
	limestone	145.85/152.91/147.08/118.65	1.06/1.02/1.08/0.90

**Table 6:** Extrapolated 28-day values of the elastic Young’s modulus, $E_{28d}$, and the creep modulus, $E_{c,28d}$.

Cement	Aggregates	$E_{28d}$ [GPa]	$E_{c,28d}$ [GPa]
CEM II/A-M (S-L) 42.5 N	quartz	38.04/40.73/35.11/34.35	163.1/177.2/148.6/137.8
	limestone	36.08/37.57/36.69/29.79	147.9/159.3/155.5/104.3
CEM II/A-S 42.5 R	quartz	37.57/39.14/36.27/42.01	165.8/167.1/153.5/181.3
	limestone	37.20/42.16/34.60/35.04	167.0/185.5/139.0/133.1
CEM I 52.5 R	quartz	42.23/43.20/38.78/36.97	208.2/204.3/179.6/154.5
	limestone	40.79/43.42/39.02/37.02	190.5/194.5/189.3/148.4

**Table 7:** Optimal dimensionless parameters $s_{E_{c}}$ (see Equation (16)).

Cement	Aggregates	$s_{E_{c}}$	${\bar{s}}_{E_{c}}$
CEM II/A-M (S-L) 42.5 N	quartzite	0.65/0.67/0.60/0.54	0.62
	limestone	0.64/0.66/0.60/0.59	
CEM II/A-S 42.5 R	quartzite	0.63/0.60/0.63/0.57	0.61
	limestone	0.63/0.61/0.65/0.54	
CEM I 52.5 R	quartzite	0.53/0.40/0.50/0.48	0.50
	limestone	0.54/0.55/0.58/0.45	

**Table 8:** Normalized evolutions of the cylinder compressive strength, Young’s modulus, and the creep modulus, quantified based on the evolution and correlation Formulas (1)–(3), (16) and (17).

	CEM II/A-M (S-L) 42.5 N			CEM II/A-S 42.5 R			CEM I 52.5 R		
**Age (Days)** t **(%)**	$\frac{f_{c}\left(t\right)}{f_{c,28d}}$ **(%)**	$\frac{E_{u}\left(t\right)}{E_{u,28d}}$ **(%)**	$\frac{E_{c}\left(t\right)}{E_{c,28d}}$ **(%)**	$\frac{f_{c}\left(t\right)}{f_{c,28d}}$ **(%)**	$\frac{E_{u}\left(t\right)}{E_{u,28d}}$ **(%)**	$\frac{E_{c}\left(t\right)}{E_{c,28d}}$ **(%)**	$\frac{f_{c}\left(t\right)}{f_{c,28d}}$ **(%)**	$\frac{E_{u}\left(t\right)}{E_{u,28d}}$ **(%)**	$\frac{E_{c}\left(t\right)}{E_{c,28d}}$ **(%)**
1	39	62	26	46	68	27	68	82	34
2	55	74	43	61	78	43	78	88	50
3	64	80	53	69	83	53	83	91	60
4	70	83	60	74	86	61	86	93	66
5	74	86	65	78	88	66	88	94	71
6	77	88	70	81	90	70	90	95	75
7	80	90	73	84	91	74	91	96	78
14	91	96	88	93	96	88	96	98	90
28	100	100	100	100	100	100	100	100	100
